# Functional Ability and Health Problems of Stroke Survivors: An Explorative Study

**DOI:** 10.7759/cureus.33375

**Published:** 2023-01-04

**Authors:** Geetha Poomalai, Suhas Prabhakar, Nalini Sirala Jagadesh

**Affiliations:** 1 Department of Nursing Foundation, Sri Ramachandra Institute of Higher Education and Research, Chennai, IND; 2 Department of Ophthalmology, Sri Ramachandra Institute of Higher Education and Research, Chennai, IND; 3 Department of Obstetrics and Gynaecology, Sri Ramachandra Institute of Higher Education and Research, Chennai, IND

**Keywords:** rehabilitation, health problems, stroke assessment, functional ability, stroke survivors

## Abstract

Background

A stroke is an emergency medical condition that needs to be treated promptly. Patients who suffer from stroke frequently experience varying degrees of impairment, necessitating emergency hospital treatment and prolonged home care. It can lower the quality of life which leads to social isolation and makes it harder to function independently. The purpose of this research was to assess the health issues and functional capacity of individuals living with stroke.

Methodology

An exploratory study was conducted in the neurological outpatient department of tertiary care hospitals in Chennai. A total of 30 post-stroke participants were selected using a convenient sampling technique. Data were collected by structured interviews using the Post-Stroke Checklist and Barthel Index. The data were analyzed through descriptive and inferential statistics.

Results

The majority of the patients were (86.7%) men in the age group of 55-65 years. Regarding the health problems identified with the Post-Stroke Checklist, the activities of daily living (80%) were the most common, and spasticity (48%) and pain (34%) were the least common. However, 60% of the participants had new problems related to vision, 66% had problems with hearing, 76% had problems with getting around inside or outside, and 60% had a history of a recent fall. Further, 52% had problems with remembering and concentrating on things, 72% had problems sleeping, and 45% were worried about their relationship with their spouse after the stroke. The median Barthel Index score was 43.5.

Conclusions

More than half of all stroke survivors were dependent on others for everyday activities. We recommend that a well-designed and focused assessment is needed to identify the functional ability and stroke-related health problems among individuals by all healthcare professionals for the successful rehabilitation of stroke survivors.

## Introduction

Stroke is a global health problem that needs immediate medical attention and prompt treatment. The degree of the brain injury and the specific brain circuits that are disrupted determine the level of impairment that a stroke patient is suffering and the level of therapy that is required. After a stroke, the brain has the innate ability to rearrange its connections from months to years to enhance function [1,2]. The needs of these patients can be complex according to the temporal phase of their illness, the cause and severity of their stroke, and other factors, including the presence of other chronic health conditions [3]. Globally, 60% of stroke patients develop permanent disabilities and experience limitations in mobility, vision, speech, and swallowing function. Literature suggests that natural recovery occurs in 50% of people, mostly in the first month with minimal recovery occurring after six months [4]. Visual loss following a stroke can be temporary or permanent and interferes with an individual’s ability to perform daily living activities and live independently [5,6]. It is strongly connected with the success of rehabilitation and can dramatically affect daily functioning. After a stroke, loss of vision can occur due to damage to the optic nerve and can lower the quality of life of patients. It leads to social isolation because it makes it harder for patients to navigate their surroundings [7,8].

According to the National Institute of Neurological Disorders and Stroke and post-stroke rehabilitation facts, proper directions and well-focused practices are the most essential components of a neurorehabilitation program [9]. It needs to be tailored to work on the stroke-related skills that are affected, such as weakness, poor coordination, difficulty walking, loss of sensation, issues with hand grasp, vision loss, and difficulty speaking or understanding [10]. Proper screening after stroke and the impact on visual symptoms can prevent further vascular injury [11].

Additionally, rehabilitation offers innovative techniques to make up for any residual limitations. Patients seem to benefit from choosing the optimal rehabilitation plan based on their capability. Patients’ individual motivational attitudes and beliefs have a significant impact on the neurorehabilitation process. Identifying care needs in frail, elderly adults, avoiding recurrent stroked, and enhancing functional outcomes and health-related quality of life after a stroke through assessments with a multidisciplinary approach are crucial [12]. The 11-item Post-Stroke Checklist may help individuals and aid medical professionals in recognizing health issues and offering advice. The Post-Stroke Checklist has been shown to be practical for people who live alone [13].

This study aimed to assess the post-stroke severity of health issues and the Barthel Index functional ability to determine the health issues and the functional ability of the post-stroke survivors.

## Materials and methods

An exploratory study was conducted in the neurological outpatient department of tertiary care hospitals in Chennai. A total of 30 individuals who were diagnosed with stroke within three to six months were selected by a convenience sampling method. This study obtained approval from the Institutional Ethical Committee of Sri Ramachandra Institute of Higher Education and Research, Chennai, and received authorization to perform the research from relevant authorities. With help from the outpatient unit staff nurses and after reviewing their records, the qualified samples were identified. Patients were explained about the objectives of the study and their freedom to participate or withdraw from it. Informed written consent was acquired from all included patients.

The participants admitted to the post-stroke rehabilitation unit were included in this study. Using the structured interview technique, baseline data on demographic variables were gathered while maintaining confidentiality. The investigation was conducted following strict ethical standards. The tools testing took between 25 and 30 minutes, during which the patients received assistance and were requested to complete the Post-Stroke Checklist for health issues and the Barthel Index questionnaire to explore their functional ability after stroke.

The health issues of the patients related to stroke were identified using the Post-Stroke Checklist. It helped assess factors that can affect the quality of life, activities of daily living, secondary prevention, mobility, spasticity, pain, incontinence, communication, mood, cognition, life after stroke, and connections with family members. Following yes/no answers for each item on the checklist, there were suggestions for the best course of action. The Barthel Index was utilized to evaluate individual autonomy in self-care. Total scores varied from 0 to 100, with 100 representing self-care independence. The additional clinical indicators and factors from the patient’s case file were noted. Descriptive and inferential statistics were used to analyze the ordinal data using SPSS software version 22.0 (IBM Corp., Armonk, NY, USA).

## Results

The mean age of the sample was 63.5 years and the majority of them 86.7% were males. Out of the 30 samples, 19 were diagnosed to have a stroke within three months or less. About 57% had an ischemic type of stroke and 47% had a hemorrhagic stroke. Overall, the majority (80%) had a hospital stay for more than 15 days, 87% were using a wheelchair for mobility, around 73% had visual impairment, and only 60% had difficulty swallowing following the stroke attack. All patients (100%) were living with assisted care at home, and 80% of the patients had cognitive impairment. Only 60% of the patients had a comorbid illness of endocrine and cardiovascular disorders (Table 1).

**Table 1:** Clinical variables of the patients with stroke (n = 30).

Variables	Frequency	%
Mean age in years	63.5 ± 1.2	
Gender		
Male	26	86.7
Female	4	13.3
Duration of stroke		
Within three months	19	63.3
Stroke type		
Hemorrhagic stroke	13	43
Ischemic stroke	17	57
Stroke location		
Right	14	47
Left	6	20
Bilateral	10	33
Stroke-related outcomes		
Length of hospital stay more than 15 days	24	80
Wheelchair use at discharge	26	87
Swallowing problems	18	60
Stroke-related visual impairment	22	73
Assisted care at home	30	100
Comorbidities		
Previous stroke (more than three months)	3	10
Dementia	16	53
Cognitive impairment	24	80
Other comorbidities - cardiovascular, diabetes, and cancer	18	60

Activities of daily living (80%), mobility problems (90%), mood swings (78%), and cognitive changes (76%) were the most prevalent health problems noted by the patients using the checklist, whereas spasticity (48%), pain (34%), and incontinence (13%) were the least prevalent. Despite the family members’ recent stroke diagnosis, 72% of the patients had changes in their relationship with them. Following the stroke attack, 66% had developed problems related to hearing, 60% reported visual impairment, 68% had trouble sleeping, and 56% had an imbalanced gait and frequent history of falls following the stroke (Table 2).

**Table 2:** Stroke-related health problems (n = 30).

Health problems after stroke	N	%
Activities of daily living	24	80
Mobility	27	90
Spasticity	14	48
Pain	10	34
Incontinence	04	13
Communication	25	82
Mood changes	23	78
Cognitive changes	23	76
Changes in life after a stroke	14	48
Relationship with family	22	72
Problems with hearing	20	66
Problems with vision	23	78
Problems with sleeping	21	68
Gait impairment and fall	17	56

**Figure 1:**
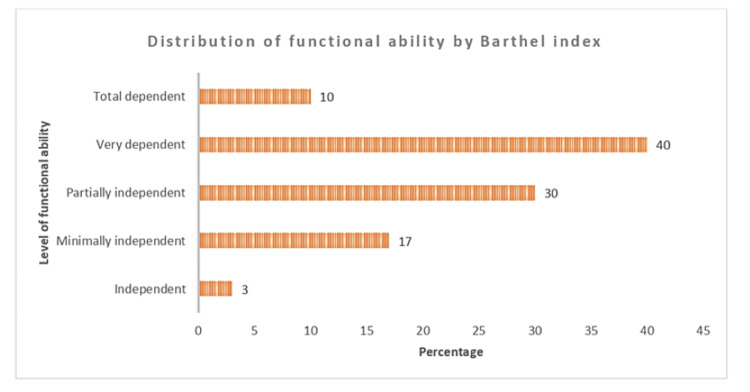
Distribution of functional ability of stroke survivors by Barthel Index.

The majority (30%) of the patients were partially independent, 40% were very dependent, and 10% were totally dependent. Out of the 30 patients, only 3% were independent, and 17 were minimally independent. The average Barthel Index score was 43.5 which showed that men’s dependency levels were higher (40) than women’s (Figure 1).

## Discussion

The Post-Stroke Checklist identified several stroke-related health issues that the patients were experiencing and that necessitated more appropriate neurorehabilitation. Most patients had a lack of knowledge about the issues and an inadequate understanding of stroke. Rehabilitation nurses must be enabled to overcome these issues as a first step by providing better treatment [13]. The abilities required to do the fundamental daily activities can be relearned by a stroke victim with the assistance of rehabilitation nurses. They also offer information on basic medical procedures, including how to take medications as prescribed, take care of the skin, deal with bladder and bowel problems, get out of bed and into a wheelchair, and special requirements for comorbidity sufferers [14,15].

Based on the Post-Stroke Checklist, this study found that patients who needed post-stroke care had a median of six health problems each. This outcome demonstrated the need for a thorough follow-up [16]. A median of three to four issues per resident was observed in earlier studies in community-dwelling groups. The significant level of reliance, comparable with earlier investigations, revealed that activities of daily living (82%) were the most often recognized health issues. Additionally, participants reported experiencing discomfort (34%) and mood disorders (78%), which is in line with earlier studies. Finding long-term needs is crucial as a result of the decline in activities of daily living during the subacute phase and the fact that individuals are frequently less active at home [17].

The post-stroke follow-ups that are conducted in a hospital setting, where a nurse is in charge and each resident has a physician, are most likely to be beneficial to stroke survivors. All patients believed that recovery required rehabilitation. Patients with high levels of drive were also more likely to comprehend rehabilitation, particularly the specialized role of the nursing staff. Earlier studies from the United Kingdom reported that almost half of the stroke patients who had decreased quality of life complained about visual impairment [18].

The majority of the participants listed achieving home independence as a personal goal, but few patients with poor motivation connected this goal to success in rehabilitation. The motivation was boosted by professional advice regarding rehabilitation, good comparisons to other stroke victims, and the desire to be discharged from the hospital. However, excessive protection from family members and specialists, a lack of knowledge, or receiving mixed messages from professionals also influenced patients’ independence [10,19].

The majority of the patients with high motivation were more likely to comprehend the idea of rehabilitation and the nursing staff’s specialized duty. In contrast to the few low-motivation patients who connected this objective to success in rehabilitation, several listed home independence as a personal goal [20]. The majority (78%) of the surveyed patients claimed that receiving knowledge about rehabilitation from experts, their encouragement of activities, their physical presence, the nursing staff’s specific interests, approaches, and the desire to leave the hospital had a positive impact on motivation. The urge to leave for home as soon as feasible positively impacted motivation [21].

About 12% of the patients said that they lacked motivation and thought that the trained nursing staff or other medical personnel should handle everything and they were concerned about their work’s performance in front of nursing staff, their encouragement of demanding tasks, and unclear information about rehabilitation from specialists. Other claimed reasons were reluctance to leave the hospital owing to a lack of a caretaker at home and bad comparisons with other patients [22].

This study’s key strength was that it showed a wide spectrum of stroke-related issues in a population that is frequently left out of the research. We made an effort to get the best patients who frequently have trouble explaining their needs. This study had a number of drawbacks. For instance, the checklist was able to elicit the existence of health problems rather than their severity as there was little input from the inhabitants, and the sample size was small because of the elaborate study logistics.

## Conclusions

More than 66% of the patients in this survey had more than six health issues. The rehabilitation of stroke patients remains the focus of the healthcare delivery system, and it can be enhanced by the active involvement of the healthcare team, the community, and family health nursing services. We recommend that a well-designed and focused assessment is needed to identify the functional ability and stroke-related health problems among individuals by all healthcare professionals for successful home-based rehabilitation of stroke survivors.
